# Investigating genetic links between blood metabolites and preeclampsia

**DOI:** 10.1186/s12905-024-03000-7

**Published:** 2024-04-05

**Authors:** Qiannan Lin, Siyu Li, Huiyan Wang, Wenbo Zhou

**Affiliations:** 1 Department of Obstetrics and Gynecology, Changzhou maternal and Child Health Care Hospital, Changzhou Medical Center, Nanjing Medical University, NO.16 Dingxiang Road, Changzhou, Jiangsu Province 213000 China; 2 Medical Research Center, Changzhou maternal and Child Health Care Hospital, Changzhou Medical Center, Nanjing Medical University, NO.16 Dingxiang Road, Changzhou, Jiangsu Province 213000 China

**Keywords:** Metabolites, Preeclampsia, GWAS, SNP, Mendelian randomization

## Abstract

**Background:**

Observational studies have revealed that metabolic disorders are closely related to the development of preeclampsia (PE). However, there is still a research gap on the causal role of metabolites in promoting or preventing PE. We aimed to systematically explore the causal association between circulating metabolites and PE.

**Methods:**

Single nucleotide polymorphisms (SNPs) from genome-wide association study (GWAS) of 486 blood metabolites (7,824 participants) were extracted as instrumental variables (*P* < 1 × 10^− 5^), GWAS summary statistics for PE were obtained from FinnGen consortium (7,212 cases and 194,266 controls) as outcome, and a two-sample Mendelian randomization (MR) analysis was conducted. Inverse variance weighted (IVW) was set as the primary method, with MR–Egger and weighted median as auxiliary methods; the instrumental variable strength and confounding factors were also assessed. Sensitivity analyses including MR-Egger, Cochran’s Q test, MR-PRESSO and leave-one-out analysis were performed to test the robustness of the MR results. For significant associations, repeated MR and meta-analysis were performed by another metabolite GWAS (8,299 participants). Furthermore, significantly associated metabolites were subjected to a metabolic pathway analysis.

**Results:**

The instrumental variables for the metabolites ranged from 3 to 493. Primary analysis revealed a total of 12 known (e.g., phenol sulfate, citrulline, lactate and gamma-glutamylglutamine) and 11 unknown metabolites were associated with PE. Heterogeneity and pleiotropy tests verified the robustness of the MR results. Validation with another metabolite GWAS dataset revealed consistency trends in 6 of the known metabolites with preliminary analysis, particularly the finding that genetic susceptibility to low levels of arachidonate (20:4n6) and citrulline were risk factors for PE. The pathway analysis revealed glycolysis/gluconeogenesis and arginine biosynthesis involved in the pathogenesis of PE.

**Conclusions:**

This study identifies a causal relationship between some circulating metabolites and PE. Our study presented new perspectives on the pathogenesis of PE by integrating metabolomics with genomics, which opens up avenues for more accurate understanding and management of the disease, providing new potential candidate metabolic molecular markers for the prevention, diagnosis and treatment of PE. Considering the limitations of MR studies, further research is needed to confirm the causality and underlying mechanisms of these findings.

**Supplementary Information:**

The online version contains supplementary material available at 10.1186/s12905-024-03000-7.

## Introduction

Preeclampsia (PE), a condition characterized by new-onset hypertension and proteinuria or other end-organ damage after 20 weeks of gestation, is a major cause of short- and long-term neonatal and maternal morbidity and mortality, with a global prevalence of 3-8% [1, 2]. As a “major obstetric syndrome”, the pathologic process of PE activates a common pathway consisting of endothelial cell activation, intravascular inflammation, and syncytiotrophoblast stress etc. [3]. Because the pathogenesis of PE has not yet been clarified and traditional methods are not yet capable of accurately predicting and treating PE at an early stage, clinical interventions remain passive after the onset of symptoms, although delivery can resolve most signs and symptoms; however, PE can persist after delivery and, in some cases, can develop de novo in the postpartum period [4, 5]. Thus, studies aiming to explore the development of new targets for disease diagnosis and treatment are important.

Compared with upstream proteomics and genomics, metabolomics is characterized by end effects and amplification effects, and its response to the physiological state of disease in organisms is more direct and sensitive, which provides a useful tool for revealing the biological mechanisms of complex diseases [6]. Metabolomics is a discipline of systematic qualitative and quantitative analyses of metabolite changes in perturbed organisms, which has a wide range of applications in the early diagnosis of diseases, interpretation of pathology and drug development [7]. As PE is a multifactorial disease, high-throughput techniques that identify multiple markers simultaneously may be an effective screening method for biomarker discovery rather than the traditional tools for detecting individual markers, and metabolomics clearly seems to be the more compatible solution [8]. Understanding the pathological process of PE development from the perspective of metabolites and discovering potential molecular markers are of great significance in all aspects of the diagnosis, evaluation, treatment and prognosis of PE. Several metabolomics studies of PE have been conducted, and metabolite profiling may be helpful in the diagnosis of preeclampsia and identification of its subtypes, with lipid metabolism and amino acid metabolic pathways being the current hotspots of research [8]. The levels of some metabolites in different pathways, such as fatty acids, carbohydrates, and amino acids, have been found to differ in patients with PE, such as uric acid, 2-oxoglutarate, taurine, alanine, glutamate, creatinine, and carnitine [9]. Another review found that a large number of metabolite pathways, such as arginine biosynthesis, phenylalanine metabolism, the citric acid cycle, and sphingolipid metabolism, are strongly associated with renal dysfunction, insulin resistance, lipid metabolism disorders, inflammatory activation, and impaired nitric oxide production, which are likely to contribute to the progression of PE [10]. For example, decanoylcarnitine, octenoylcarnitine, 3-hydroxyisovaleric acid, and xanthine showed a sustained upward trend, whereas succinate, formate, and taurine showed a sustained downward trend in PE patients compared with controls [10]. These studies have identified many metabolic biomarkers that may be useful for PE risk prediction and clinical diagnosis. However, the current studies have some limitations, such as high variability (e.g., diet, lifestyle, analytical and experimental conditions, and data analysis methods), which may affect the final results and lead to controversy about the association of some metabolites with PE [7]. Furthermore, the causal association of these metabolites with PE has not been elucidated.

Mendelian randomization (MR) is an epidemiological research methodology that uses large genome-wide association study (GWAS) datasets to extract genetic tools unaffected by acquired confounders to infer causal relationships between exposures and outcomes and can be used to complement evidence from observational and clinical studies; thus, MR is thought to be less subject to measured and unmeasured confounders [11, 12]. With the development of specific research techniques (e.g., genome-wide association studies (GWAS), epigenetics, and metabolomics), MR analyses have been widely used for inferring causality of various exposures and outcomes [13]. GWAS can provide molecular insights into the complex interactions between environmental and genetic factors in disease pathogenesis; moreover, a large number of single nucleotide polymorphisms (SNPs) have been identified as strongly correlated with serum metabolites [14]. Several MR studies have explored the causal association of metabolites with diseases [15–17]. In this study, we aimed to systematically explore the causal association between circulating metabolites and PE, which will provide a new molecular theoretical basis and a new potential target for the diagnosis and treatment of the development of PE.

## Materials and methods

### Study design

In this study, we explored the causal associations of these genetically predicted metabolites with PE by performing two-sample MR using publicly available GWAS summary statistics, serum metabolite-associated SNPs as instrumental variables, and GWAS data from patients with PE as outcomes. Metabolites preliminarily screened for significant causal associations were subjected to an inverse MR analysis and further validated using another metabolite GWAS dataset. Finally, a pathway analysis of these metabolites was performed. These GWAS summary data were derived from publicly available datasets and therefore did not require additional ethical approval.

### Exposure and outcome GWAS data

The GWAS for the exposures analyzed in this study as a preliminary assessment were derived from the meta-study reported by Shin et al., which included the plasma or serum from 7,824 adult individuals from two European population studies, the TwinsUK cohort (mean 53 years) and KORA (mean 61 years), and revealed significant associations at 145 metabolic loci. After stringent quality controls, a subset of 486 metabolites was available for genetic analysis in both cohorts, including 309 known and 177 unknown metabolites [14]. Another GWAS dataset of serum metabolites used for validation was derived from the recently published study by Chen et al. [18]. This study comprises 1,091 metabolites and 309 metabolite ratios in 8,299 individuals from the Canadian Longitudinal Study on Aging (CLSA) cohort, identifying associations with 690 metabolites at 248 loci. Although individuals from other ethnic groups, such as individuals of African and East Asian ancestry, were also included in the study by Chen et al., in this study, we focused on and included only individuals of European ancestry to be consistent with the source of the final dataset. All of these known metabolites were divided into eight broad metabolic groups, including amino acid, carbohydrate, cofactors and vitamin, energy, lipid, nucleotide, peptide and xenobiotic metabolism. GWAS data for PE as an outcome were derived from the latest release of R9 data from the FinnGen consortium (https://www.finngen.fi/en/access_results), and a total of 7,212 patients and 194,266 normal control individuals were included. The individuals included were all of European ancestry.

### Instrumental variable (IV) selection

Considering the limited number of SNPs that reach genome-wide significance, as a method to include more metabolites in the analysis, which reported by other published studies [19, 20], the criteria for IVs associated with genetically determined blood metabolites used in this study are as follows: *P* < 1 × 10^− 5^; linkage disequilibrium (LD), R^2^ < 0.1; and clump distance = 500 kb. These IVs must be reproducibly and strongly associated with the exposure; they must not be associated with confounders (i.e., factors that confound the relationship between the exposure and outcome); and must only associated with the outcome through the exposure (i.e., they are independent of the outcome given the exposure) [21]. In the follow-up analysis, we performed an MR analysis after further excluding SNPs associated with potentially relevant confounders of PE using the PhenomenonScaner website (http://www.phenoscanner.medschl.cam.ac.uk/), such as diabetes mellitus, body mass index (BMI), obesity, diastolic blood pressure, systolic blood pressure, and hypertension etc. [22]. For these IVs, we also performed F value statistics with a value greater than 10 to effectively avoid weak instrumental variable bias. When harmonizing the exposure and outcome data, we tried to infer positive strand alleles using allele frequencies for palindromes; if the exposed SNP did not exist in the outcome GWAS dataset, we used a proxy SNP (R^2^ > 0.8).

### MR analysis

Inverse variance weighted (IVW) was used as the main primary screening method, with MR–Egger and weighed median as auxiliary methods. In the IVW hypothesis, IVs were considered to have no pleiotropy, and the combined effect was assessed by calculating the Wald ratio for each IV, with the corresponding inverse variance used as a weight in the meta-analysis [23]. In the MR–Egger hypothesis, the intercept is used to assess polytropy. If this intercept is very close to 0, then the MR‒Egger regression model is very close to the IVW, but if the intercept is very different from 0, then it suggests that horizontal pleiotropy may exist between these IVs [24]. The median of the distribution function obtained using weighed median is obtained by sorting all IV effect values according to their weights, and weighed median obtains robust estimates when at least 50% of IVs are valid [25]. In the sensitivity analysis, through the MR pleiotropy residual sum and outlier (MR–PRESSO) global test the IVW result was calculated for each SNP after removing that SNP, and then the residual sum of squares of the effect of that SNP and the IVW result was calculated. If the P value for the global test is greater than 0.05, horizontal pleiotropy exists in the IVs. We therefore removed significant outliers using MR–PRESSO to reduce polyvalence and obtain more accurate estimates [26]. IVW Cochran’s Q-statistical test was used as a test for heterogeneity, and the MR–Egger intercept was used as a horizontal multiple validity test. Finally, each IV was removed individually using the leave-one-out method, and the MR results for the remaining SNPs were calculated to verify whether the MR results would be strongly perturbed after the removal of specific IVs.

### Metabolic pathway analysis

A metabolic pathway analysis of known metabolites with potential causal associations was performed based on the MR analysis results. The online analytics are based on MetaboAnalyst 5.0 (https://www.metaboanalyst.ca/).

### Statistical analysis

The MR analysis was implemented using the “TwoSampleMR” package (version 0.5.7) based on R (version 4.2.3). MR–PRESSO was performed using the “MRPRESSO” package (version 1.0). Given that a total of 486 metabolites were investigated in the present study, we accounted for multiple testing in our analyses using a Bonferroni corrected threshold of *P* < 1.03E^− 4^ (0.05/486) as significant evidence of associations [27], and a *P* value between 0.05 and 1.03E^− 4^ was considered suggestive evidence of associations. The effects of candidate blood metabolites were further validated by selecting metabolites that were causally associated with PE in the preliminary analyses as exposures in another independent metabolite GWAS dataset, and MR analyses were performed as described above for further validation. The combined effects were assessed using either a random or a fixed-effects IVW model meta-analysis based on the R package.

## Results

### Results from the MR analysis reveal metabolites causally associated with PE

**Fig. 1:**
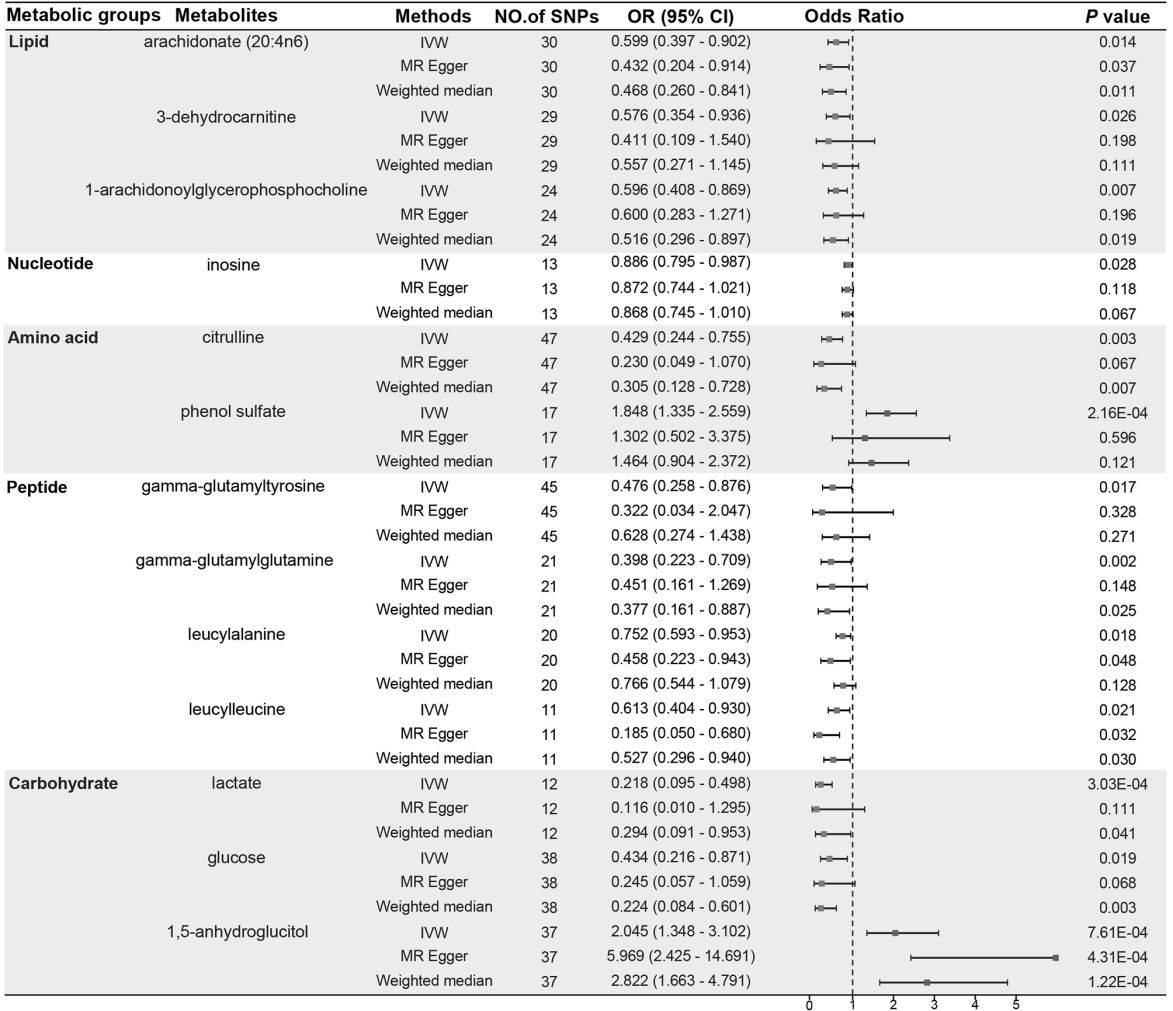
MR estimates of known metabolites on the risk for PE IVW: inverse variance weighted; SNP: single nucleotide polymorphism; OR: odds ratio; CI: confidence interval; MR: Mendelian randomization; PE: preeclampsia

The instrumental variables (IVs) for the metabolites ranged from 3 to 493, The F statistics of all SNPs associated with metabolites were greater than 10, indicating absence of weak IVs. The IVW method suggested that a total of 30 metabolites with *P* < 0.05 were present among the 486 metabolites. Combining MR–Egger and weighed media methods, 26 metabolites had consistent directions, which included 3 lipids, 1 nucleotide, 2 amino acids, 4 peptides, 3 carbohydrates, and 13 unknown metabolites, while the other 4 metabolites with inconsistent directions were excluded. Detailed information on the genetic IVs for these 26 metabolites is shown in Tables S1 and S2. The known metabolites that were causally associated with PE were three lipids: arachidonate (20:4n6) (OR = 0.599, 95% CI = 0.397–0.902, *P* = 0.014), 3-dehydrocarnitine (OR = 0.576, 95% CI = 0.354–0.936, *P* = 0.026) and 1-arachidonoylglycerophosphocholine (OR = 0.596, 95% CI = 0.408–0.869, *P* = 0.007); one nucleotide: inosine (OR = 0.886, 95% CI = 0.795–0.987, *P* = 0.028); two amino acids: citrulline (OR = 0.429, 95% CI = 0.244–0.755, *P* = 0.003) and phenol sulfate (OR = 1.848, 95% CI = 1.335–2.559, *P* = 2.16E^− 4^); four peptides: gamma-glutamyltyrosine (OR = 0.476, 95% CI = 0.258–0.876, *P* = 0.017), gamma-glutamylglutamine (OR = 0.398, 95% CI = 0.223–0.709, *P* = 0.002), leucylalanine (OR = 0.752, 95% CI = 0.593–0.953, *P* = 0.018) and leucylleucine (OR = 0.613, 95% CI = 0.404–0.930, *P* = 0.021); and three carbohydrates: lactate (OR = 0.218, 95% CI = 0.095–0.498, *P* = 3.03E^− 4^), glucose (OR = 0.434, 95% CI = 0.216–0.871, *P* = 0.019) and 1,5-anhydroglucitol (OR = 2.045, 95% CI = 1.348–3.102, *P* = 7.61E^− 4^). Thirteen unknown metabolites were also causally associated with PE, five of which were causal risk factors for PE, and the remaining eight were protective factors for PE. The forest plots in Fig. 1 and Figure S1 show all known and unknown metabolites that were causally associated with PE.

Subsequent sensitivity analyses (Table 1) revealed potential pleiotropy for IVs of the metabolite 1,5-anhydroglucitol (MR–Egger intercept *P* = 0.014), while the MR–PRESSO global test and the IVW Cochrane’s Q method suggested the presence of heterogeneity (*P* < 0.05), implying that causal associations may not be robust. In contrast, the IVs of the other 12 known and 13 unknown metabolites did not exhibit heterogeneity or pleiotropy (Table S3). The leave-one-out method also suggested that these metabolite-associated outcomes were not driven by a single SNP (Figures S2 and S3).

**Table 1:** Results of pleiotropy and heterogeneity test of the known metabolites on PE

		Pleiotropy test				Heterogeneity analyses		
Metabolic groups	Metabolites	MR- Egger Intercept	se	*P*	MR- PRESSO global test *P*		IVW Cochrane’s Q	Q *P* value
Lipid	arachidonate (20:4n6)	0.007	0.007	0.317	0.647		25.949	0.628
	3-dehydrocarnitine	0.007	0.012	0.594	0.710		23.439	0.711
	1-arachidonoylglycerophosphocholine	-1.69E-04	0.008	0.983	0.591		21.476	0.552
Nucleotide	inosine	0.003	0.010	0.788	0.947		5.033	0.957
Amino acid	citrulline	0.007	0.008	0.397	0.584		43.805	0.565
	phenol sulfate	0.015	0.019	0.455	0.829		10.889	0.816
Peptide	gamma-glutamyltyrosine	0.004	0.011	0.724	0.163		52.792	0.171
	gamma-glutamylglutamine	-0.003	0.009	0.773	0.447		21.567	0.364
	leucylalanine	0.022	0.016	0.171	0.553		17.564	0.552
	leucylleucine	0.035	0.019	0.089	0.487		10.034	0.437
Carbohydrate	lactate	0.010	0.019	0.596	0.581		9.989	0.531
	glucose	0.007	0.008	0.389	0.293		41.432	0.283
	1,5-anhydroglucitol	-0.023	0.009	0.014	0.035		52.356	0.038

MR: Mendelian randomization; IVW: inverse variance weighted; MR-PRESSO: MR pleiotropy residual sum and outlier; PE: preeclampsia

### Confounding factors

Confounding factors, such as diabetes mellitus, body mass index (BMI), obesity and hypertension, have been reported to be associated with PE [28]. Although sensitivity analyses suggested no potential pleiotropy, we screened and excluded SNPs associated with the above confounders using the PhenomenonScaner website and subsequently repeated the MR analysis. Nine of the 12 IVs for known metabolites had confounder-associated SNPs (Table S4), and thus the MR analysis was performed again after removing these SNPs. The IVW method suggested that most metabolites, except for 3-dehydrocarnitine and leucylalanine, were still significantly causally associated with PE, consistent with the preliminary analysis (Table S5). Sensitivity analyses of these reanalyzed metabolite-associated IVs suggested no pleiotropy or heterogeneity (Table S6). In addition, among the unknown metabolites, we found confounder-associated SNPs in the IVs associated with metabolites X-10,510, X-11,470, X-11,787, X-12,056 and X-13,859. After removing these SNPs, the IVW method suggested that X-10,510 and X-12,056 no longer had significant causal associations with PE (Table S5). The results of sensitivity analyses suggested that after removing the confounder factor-related SNPs, the IVs of the other three metabolites did not exhibit pleiotropy or heterogeneity (Table S6).

### Analysis of a reverse causal association

We used PE from the Finnish GWAS dataset as an exposure extraction instrumental variable and two-sample reverse MR analysis with the metabolite GWAS reported by Shin et al. as the outcome to exclude potential revers causal associations. A total of 38 IVs (*P* < 5E^− 6^) were obtained (Table S7). The IVW method suggested that no significant causal associations existed between PE and any of these 12 known and 11 unknown metabolites (Table S8), and the sensitivity analyses did not reveal the presence of potential pleiotropy and heterogeneity (Table S9).

### Validation and meta-analysis of another independent metabolite GWAS dataset

**Fig. 2:**
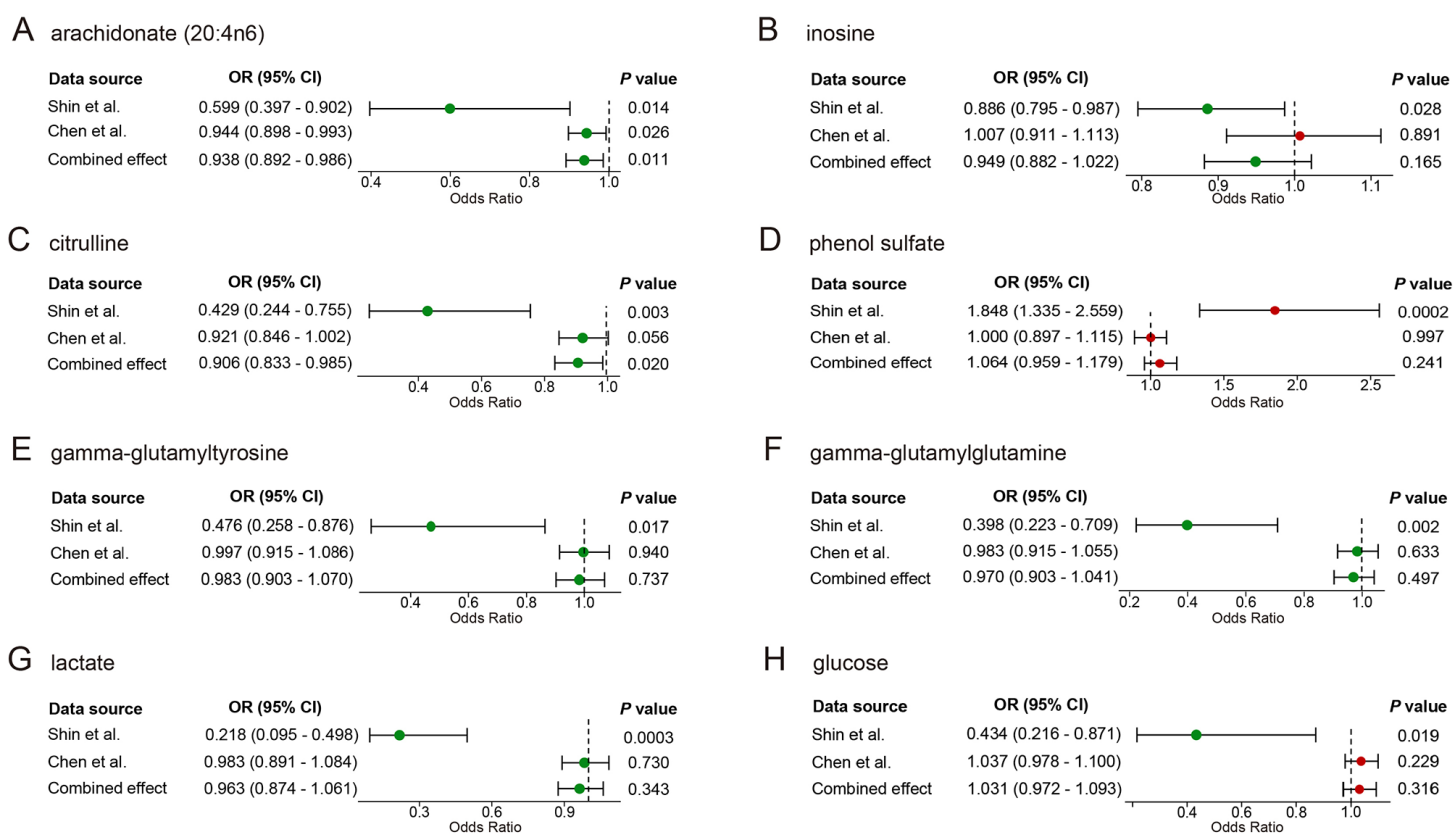
Meta-analysis of the causal relationship between metabolites with different GWAS datasets and PE estimated by IVW method OR: odds ratio; CI: confidence interval; IVW: inverse variance weighted; GWAS: genome wide association studies; PE: preeclampsia

We applied another GWAS dataset derived from the most recent publication by Chen et al., which contains 8 of the 12 known metabolites with significant associations mentioned above, to further validate the robustness of the metabolites presenting causal associations with PE explored above. We first explored the association of these metabolites with PE using the IVW method, followed by a meta-analysis with the results reported by Shin et al., which suggested that 6 of these 8 metabolites showed a consistent trend of association with PE (Fig. 2): arachidonate (20:4n6), citrulline, phenol sulfate, gamma-glutamyltyrosine, gamma-glutamylglutamine and lactate. Among these metabolites, only one had a significant association: arachidonate (20:4n6) (OR = 0.944, 95% CI = 0.898–0.993, *P* = 0.026). This finding may be result from population heterogeneity across different GWAS datasets. The combined effects of the meta-analysis also suggested that genes predicting low levels of arachidonate (20:4n6) (OR = 0.938, 95% CI = 0.892–0.986, *P* = 0.011) and citrulline (OR = 0.906, 95% CI = 0.833–0.985, *P* = 0.020) were significantly associated with a higher PE risk.

### Metabolite pathway analysis

Based on the 12 known significantly related metabolites, we performed an online metabolite pathway analysis (https://www.metaboanalyst.ca/). The results suggested that these metabolites were enriched in a total of six pathways, including glycolysis/gluconeogenesis, arginine biosynthesis, pyruvate metabolism, biosynthesis of unsaturated fatty acids, arachidonic acid metabolism and purine metabolism (Table S10). Among them, glycolysis/gluconeogenesis (*P* = 0.003) and arginine biosynthesis (*P* = 0.044) were significant enriched.

## Discussion

In this study, we explored the causal relationship between metabolites and PE by performing two-sample MR of a large metabolite GWAS dataset. The causal associations of some circulating metabolites with PE have been shown for the first time from a genetic point of view, which fills a gap in the field. In particular, through validation using another metabolite GWAS dataset, genetic variants that predicted low levels of arachidonate (20:4n6) and citrulline were found to be risk factors for PE in both datasets. In addition, the metabolite pathway analysis indicated that glycolysis/gluconeogenesis and arginine biosynthesis pathways were significantly associated with PE.

PE is a pregnancy-specific disease that can rapidly progress to severe complications, including the death of the mother and fetus, and poses a serious risk to the health of the mother and child [3]. Therapeutic options are limited at present, as the exact pathogenesis is unknown, and delivery is the only definitive treatment [29]. To date, the diagnosis of PE has focused on blood pressure and urine protein levels; however, these biomarkers are still not fully and readily available for use in clinical practice, and some of them can only be assessed in the mid- or late-gestational period with low specificity or sensitivity. Therefore, studies aiming to explore new biomarkers to improve the diagnosis and deepen our understanding of the pathogenesis of PE are needed [10]. Metabolites are the end products of transcription, translation, and proteomics and are therefore highly relevant to the functions and phenotypes of biological systems [30, 31]; they also have a wide range of applications in early disease diagnosis, pathology interpretation, and drug development. Metabolites provide more direct feedback on biological activity than genes and proteins, and even small lesions in genes, transcripts, and proteins become significant at the metabolite level, making them easy to detect [31]. Furthermore, metabolite measurements are significantly lower than those for genes and proteins in terms of the number of tests performed and the difficulty of the tests [9]. Extensive adaptive physiological changes in maternal, fetal, and placental function occur during pregnancy, and circulating maternal metabolites, such as triglycerides, cholesterol, and free fatty acids, change significantly during pregnancy to meet the energy and catabolic needs of the fetus in utero [7]. In terms of PE studies, Kelly et al. used liquid chromatography–tandem mass spectrometry to identify 72 (0.9%) metabolite profiles associated with PE, with glycerophospholipid, arachidonic acid and glycerol metabolism, and fatty acid biosynthesis being the most enriched metabolic pathways [32]. Another metabolomics review that included 41 studies identified a total of 33 high-frequency metabolites (reported in ≥ 3 studies), such as creatinine, glycine, L-isoleucine, and glucose [10]; several metabolic pathways associated with PE were also identified, in particular alanine, aspartic acid, and glutamate metabolism, and these biomarkers and pathways are strongly associated with lipid metabolism disorders, inflammation activation, impaired nitric oxide production, etc., which are likely to contribute to the progression of PE [10].

Although the literature strongly suggests a relationship between metabolic disorders and PE, the current body of evidence does not clarify the causal association of circulating metabolites with the development of PE. Furthermore, the potential causal relationship between serum metabolite profiles and PE has not been fully elucidated. The sample sizes of relevant reported studies are usually small, and the findings of some studies are inconsistent. These limitations provide an opportunity for MR, a causal inference method that uses genetic variation in large samples as an instrumental variable to test for the role of exposure in disease [11]; because alleles are randomly assigned at conception, this process of randomization typically breaks down confounding with most risk factors and reduces the tendency for confounding to bias outcomes [18, 21]. In the present study, we found that arachidonate (20:4n6), 3-dehydrocarnitine and 1-arachidonoylglycerophosphocholine, which are lipids, were negatively correlated with the risk of PE and were protective factors for PE. Arachidonate, an n-6 long-chain polyunsaturated fatty acid, plays an important role in fetal and infant growth and development [33]. Uterine spiral artery remodeling requires an inflammatory environment to repair the uterine epithelium and remove cellular debris, and immune cells, including macrophages, natural killer cells, and dendritic cells, penetrate the meconium and accumulate around the trophectoderm, a self-limiting, “physiological” inflammatory process, the absence of which results in failure of implantation and abnormal placental development [34]. Studies have shown that arachidonate plays an important role in the preparation for and during pregnancy and is strongly associated with ovulation, menstruation, pregnancy and labor, and the onset of physiological inflammatory responses [33]. No studies have reported the association of 3-dehydrocarnitine, an intermediate in carnitine degradation, with pregnancy or hypertension, and this metabolite was found to significantly reduce body weight and body mass index (BMI) and improve insulin resistance when taken with a carnitine supplement in a prospective randomized controlled trial [35]. These aforementioned factors are closely associated with the development of PE, and thus this result is consistent with the findings of this study that low levels of 3-dehydrocarnitine may be a risk factor for PE. 1-Arachidonoylglycerophosphocholine combines the structure of choline glycerophosphate with the branched chain of arachidonic acid. Glycerophosphocholine is the metabolite with the greatest discrimination between PE and the normal group in late pregnancy [36], and several studies have confirmed the association between glycerophosphorylcholine and the development of PE; however, the findings are controversial [37, 38]. These inconsistencies may result from the presence of different modifications and isoforms. Studies reporting on 1-arachidonoylglycerophosphocholine and PE are lacking, and our finding that low levels of 1-arachidonoylglycerophosphocholine are a risk factor for PE must be validated by in-depth studies.

We found that genetically predicted high citrulline levels were a protective factor for PE. The citrulline level in pregnancies with severe PE is lower than that in pregnancies without PE [39], and supplementation with citrulline also produced positive results in a mouse model of PE, which may be attributable to beneficial effects on maternal vascular health via the nitric oxide (NO) signaling pathway [39]. Another study found that citrulline supplementation improved placental insufficiency, fetal growth and endothelial function by downregulating the TLR4/NF-κB inflammatory pathway [40]. This result is consistent with our findings that citrulline is a protective factor for PE. In addition, the present study identified high levels of phenol sulfate as a risk factor for PE, and phenol sulfate is involved in phenylalanine and tyrosine metabolism [41]. The most important metabolic pathway of phenylalanine is the generation of tyrosine catalyzed by hydroxylase, which subsequently generates dopamine [42]. Some studies have confirmed that dopamine can cause oxidative stress, abnormal expression of nitric oxide and endothelin in vascular endothelial cells, vasoconstriction, and elevated blood pressure, which can lead to gestational hypertension and PE [43]. Studies confirm increased phenylalanine levels in both PE patients and newborns [44, 45]. This result was also consistent with the findings of this study. In addition, the present study also identified four amino acid dipeptide metabolites that were causally associated with PE, including gamma-glutamyltyrosine, gamma-glutamylglutamine, leucylalanine and leucylleucine, all of which are protective factors for PE. No studies have reported associations of these two metabolites with PE, requiring follow up.

Another finding of this study is that genetically predicted high levels of inosine were a protective factor for PE. Inosine is a central intermediate in the purine biosynthesis and degradation pathway and is present in noncoding and exogenous RNAs, in which it plays a key structural and functional role as an important molecular messenger in cell signaling pathways [46]. Inosine is neuroprotective in rats with spinal cord injury by scavenging free radicals [47]. Another study found that after tissue ischemia, inosine maintains homeostasis by stimulating the release of hepatic glucose via A3 adenosine receptors [48]. Therefore, we hypothesized that inosine might exert a similar protective effect under PE placental ischemic hypoxic conditions. However, inosine metabolism also produces uric acid, which is a risk factor for the development of PE [9]. Therefore, the causal association between inosine and PE still must be determined by performing subsequent in-depth studies.

Two carbohydrate metabolites, lactate and glucose, were causally associated with PE. Insulin resistance is a clear risk factor for PE, and elevated insulin levels can lead to increased sympathetic nervous system activity and renal sodium retention, which may increase blood pressure [49]. In addition, insulin is a hypoglycemic hormone that facilitates the entry of blood glucose into cells to be used for energy, and controversy about the blood glucose level in patients with PE exists [10]. Insulin resistance would affect the entry of blood glucose into cells, which could explain the elevated blood glucose levels in PE patients found in some observational studies [10]; on the other hand, however, a decrease amount of glucose entering the cell would affect the energy metabolism of the trophoblasts. Throughout pregnancy, glucose is not only present as an energy substrate but also regulates embryo implantation and placental development [50, 51]. Thus, this could partially explain the finding in the present study that reduced glucose may associated with a higher PE risk. Early in gestation, the blastocyst exhibits a rather specialized metabolism, converting 90% of the glucose consumed to lactate even with a sufficient oxygen supply for rapid proliferation and division [52]. Lactate creates a low pH microenvironment around the embryo that aids in the breakdown of uterine tissue and promotes trophoblast invasion; lactate also acts as a signaling molecule that induces VEGF recruitment from uterine cells and promotes angiogenesis [53]. This result suggests that the high lactate/low pH region produced by blastocysts modulates the activity of the local immune response and contributes to the generation of immune tolerance [53]. Clinical evidence indicates that plasma lactate levels in PE patients are lower than those in the control normal group [54, 55]. Thus, lactate is a key metabolic mediator necessary during pregnancy [50]. This is consistent with the findings of this study that low lactate levels may be associated with a higher risk of PE.

We introduced GWAS data for another metabolite to further validate the robustness of the causal associations of our identified metabolites with PE, and the results showed that six metabolites exhibited a consistent trend for the association identified in the preliminary analysis; however, only one, arachidonate (20:4n6), showed a significant association with PE. By performing a meta-analysis, we found that both arachidonate (20:4n6) and citrulline were protective factors for PE. Since the association results for the remaining metabolites in the two datasets were not consistent, their causal association with PE still needs to be determined by performing subsequent studies. Based on the metabolite pathway analysis, we found that glycolysis/gluconeogenesis and arginine biosynthesis were closely related to PE. As mentioned above, glycolysis plays an important regulatory function in placental implantation and energy supply. In addition, other studies have confirmed the correlation between arginine biosynthesis and PE [10]. Clinical and experimental data suggest that the arginine biosynthesis pathway contributes to improved NO availability in the placenta and uteroplacental circulation and reduced maternal blood pressure [56]. Therefore, this finding coincides with the current understanding of PE pathophysiology and provides new genetic insights for further research.

This study has several limitations. First, the MR analysis is not a substitute for randomized controlled studies, and differences in diet, lifestyle, etc., may affect individual susceptibility. Second, we relaxed the metabolite instrumental variable significance thresholds with reference to the common practice in the published relevant literature to include a larger number of SNPs, which may reduce the power of IVs and cause bias in the results. Although we removed confounder-associated SNPs, we did not completely exclude potential levels of pleiotropy. Third, only individuals of European ancestry were included in this study, and validation in additional populations is needed. Fourth, we assessed the association of blood metabolites with PE, and the biological functions of different metabolites may be tissue- and cell-specific. Finally, large-scale rigorous randomized controlled studies are necessary to verify the causal association of these metabolites with PE and to explore and clarify the molecular mechanisms involved in the development of PE.

## Conclusions

In conclusion, in the present MR study, we provide some preliminary evidence for a causal relationship between circulating metabolites and PE through integrating genomic and metabolomic data, which contributes to our understanding of the complex role of metabolites in PE development. Particularly, a genetic susceptibility to low levels of arachidonate (20:4n6) and citrulline is a risk factor for PE. Our study provides new candidate molecules for PE screening, prevention and mechanistic explorations in clinical practice. However, comprehensive and in-depth clinical and mechanistic studies are needed for validation.

### Electronic supplementary material

Below is the link to the electronic supplementary material.

Supplementary Material 1

Supplementary Material 2

## Data Availability

The summary statistics of blood circulating metabolites GWAS data were obtained from GWAS Catalog (https://www.ebi.ac.uk/gwas/); The summary statistics of PE GWAS data was obtained from FinnGen consortium (https://www.finngen.fi/en/access_results).

## Abbreviations

PE: preeclampsia
MR: Mendelian randomization
GWAS: genome-wide association studies
SNP: single nucleotide polymorphisms
IVW: inverse variance weighted
CLSA: Canadian longitudinal study on aging
IV: instrumental variables
LD: linkage disequilibrium
MR-PRESSO: Mendelian randomization pleiotropy RESidual sum and outlier
OR: Odds ratio
BMI: body mass index
NO: nitric oxide
