# Supplementary material for: Investigating genetic links between blood metabolites and preeclampsia
Source: BMC Womens Health. 2024 Apr 5;24:223. doi: 10.1186/s12905-024-03000-7 (PMC10996307; doi:10.1186/s12905-024-03000-7)

**Supplementary Figure S1, MR estimates of unknown metabolites on the risk for PE.**

IVW, inverse variance weighted; SNP, single nucleotide polymorphism; OR, odds ratio; CI, confidence interval; MR, Mendelian randomization; PE, preeclampsia.
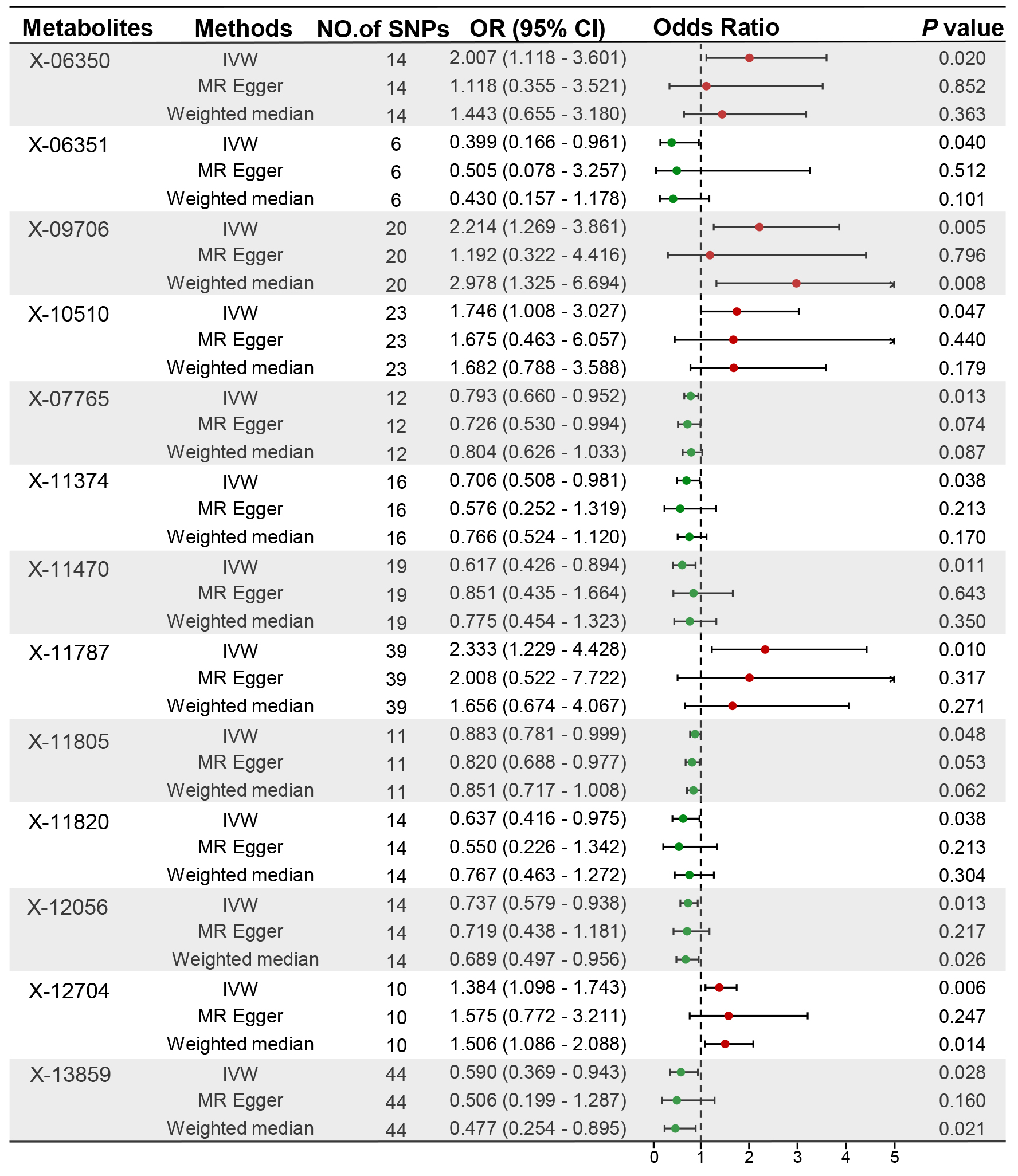


**Supplementary Figure S2, The leave-one-out analysis of known metabolites on preeclampsia when leaving one SNP out.**

SNP, single nucleotide polymorphism. The error bar represents the 95% confidence interval with inverse variance weighted method. A, arachidonate (20:4n6); B, 3-dehydrocarnitine; C, 1-arachidonoylglycerophosphocholine; D, inosine; E, citrulline; F, phenol sulfate; G, gamma-glutamyltyrosine; H, gamma-glutamylglutamine; I, leucylalanine; J, leucylleucine; K, lactate; L, glucose.


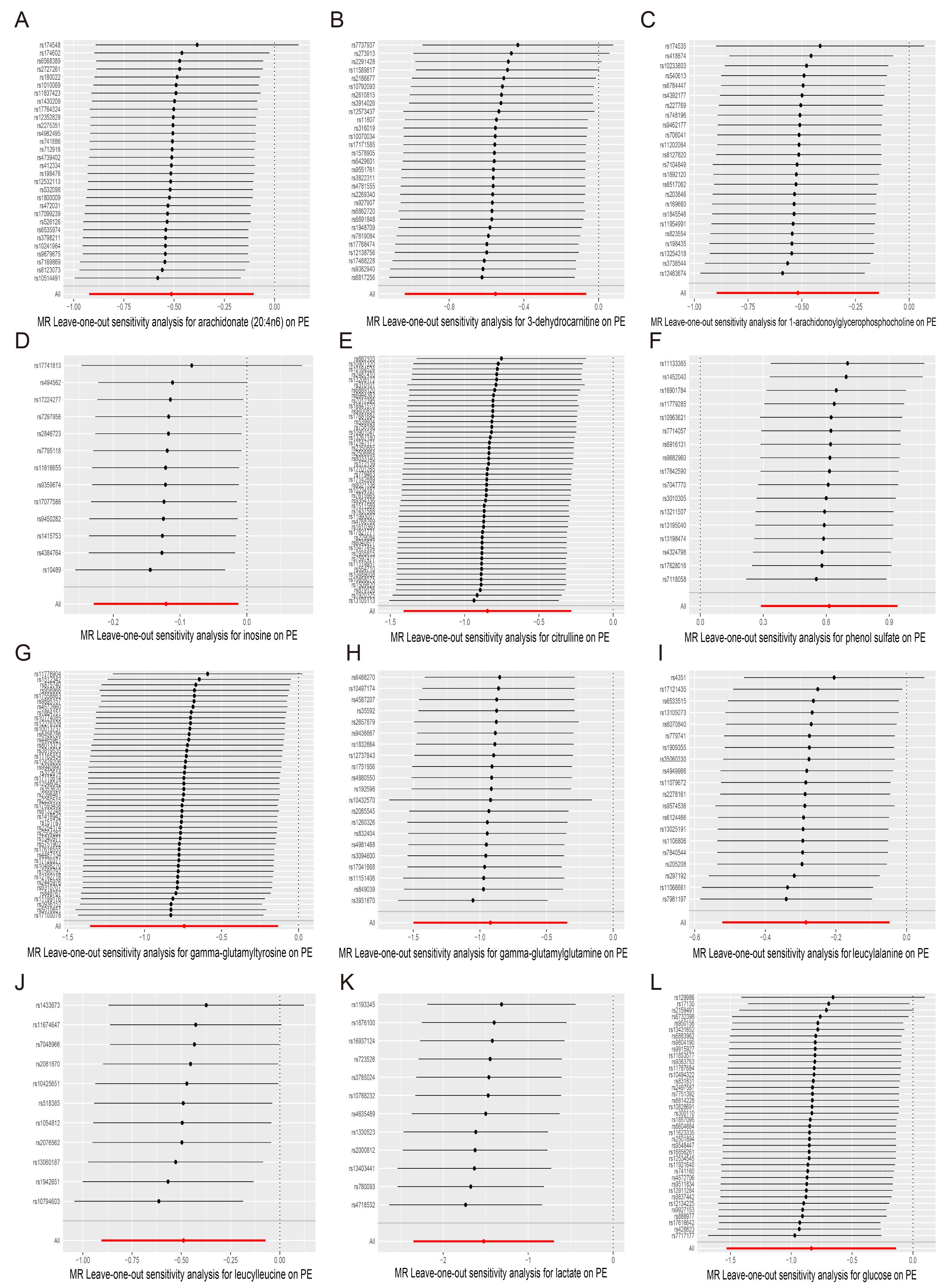


**Supplementary Figure S3, The leave-one-out analysis of unknown metabolites on preeclampsia when leaving one SNP out.**

SNP, single nucleotide polymorphism. The error bar represents the 95% confidence interval with inverse variance weighted method. A, X-06350; B, X-06351; C, X-09706; D, X-10510; E, X-07765; F, X-11470; G, X-11787; H, X-11805; I, X-11820; J, X-12056; K, X-12704; L, X-13859; M, X-11374.


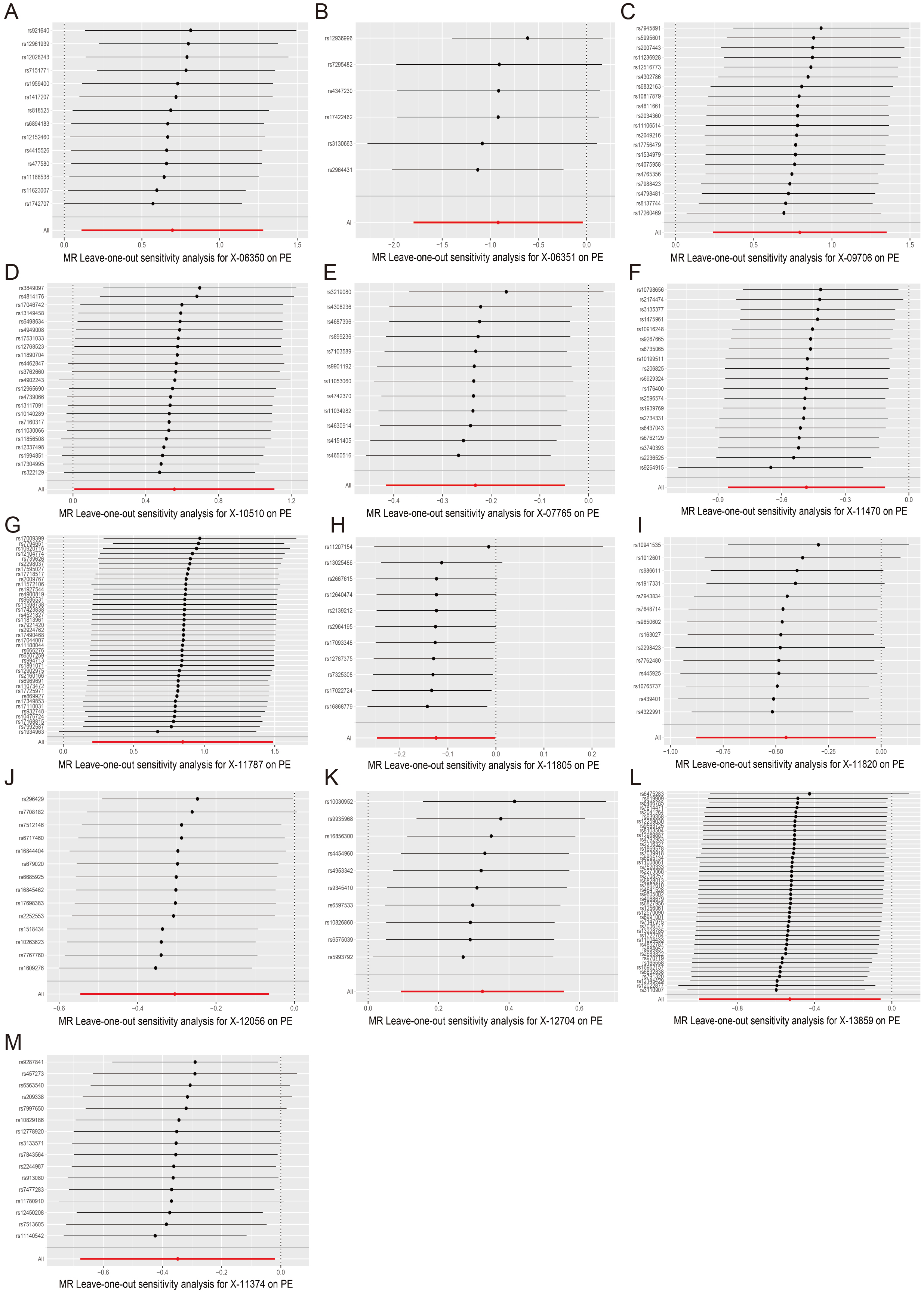

Supplement: Supplementary file 2 — Supplementary Material 2 [file 12905_2024_3000_MOESM2_ESM.docx]
